# A driver or a passenger? D421-caspase cleaved tau is associated with aggregated and insoluble tau

**DOI:** 10.1186/1750-1326-8-S1-P47

**Published:** 2013-09-13

**Authors:** Yolanda Gibson, Katya Garn, Clare Hardy, Manuela Piarulli, Sarah Glover, Mark Ward, Emily McNaughton, Michael Hutton, Michael O’Neill, Joanna Wolak

**Affiliations:** 1 Lilly Research Centre, Windlesham, UK

## Background

Aggregation of tau is one of the main hallmarks of Alzheimer’s disease and other tauopathies. Normally tau regulates microtubule (MT) dynamics and hence axonal function but in AD hyperphosphorylated, misfolded and aggregated tau has reduced interaction with microtubules. There is growing evidence that the proteolytic processing of tau may play a crucial role in the aberrant change of tau conformation and its aggregation. rTg4510 mice express the human four-repeat tau gene lacking the amino-terminal sequence (0N4R) and with a P301L mutation. Between four and ten months of age the mice develop progressive tau pathology and eventually neuronal loss. The aim of this work was to investigate the importance of tau cleavage at D421, the cleavage epitope for caspase-3 and caspase-6, and its role in tau aggregation. We ran a longitudinal study to measure the level and distribution of D421-caspase cleaved tau in rTg4510 between two and eight months of age to determine whether there is a correlation between the cleavage of tau and its conformational change.

## Material and methods

Total tau and D421-caspase cleaved tau levels in the cortex and hippocampus in each age group were quantified using a panel of proprietary AlphaScreen assays and by standard IHC methods. Tau aggregation was assessed using high-speed centrifugation of brain homogenates.

## Results

The biochemical and immunohistochemical analyses of rTg4510 showed a time-dependent increase of caspase-cleaved tau at D421 in the cortex and hippocampus between four and eight months of age and importantly caspase-cleaved tau was shown to be associated with the insoluble fraction of tau. These changes are concomitant with progression of tau pathological changes previously observed in this model.

## Conclusions

Caspase-cleaved tau at D421 is associated with aggregated and insoluble tau. Our findings contribute to the discussion on whether therapeutics aimed at inhibiting caspase-mediated tau cleavage could be beneficial in slowing cleavage and aggregation, and hence potentially inhibit the pathological cascade in Alzheimer’s disease.

